# Supplementary material for: Environmental DNA reveals that rivers are conveyer belts of biodiversity information
Source: Nat Commun. 2016 Aug 30;7:12544. doi: 10.1038/ncomms12544 (PMC5013555; doi:10.1038/ncomms12544)
Supplement: Supplementary Information — Supplementary Figures 1-3, Supplementary Tables 1-7, Supplementary Notes 1 and Supplementary References [file ncomms12544-s1.pdf]

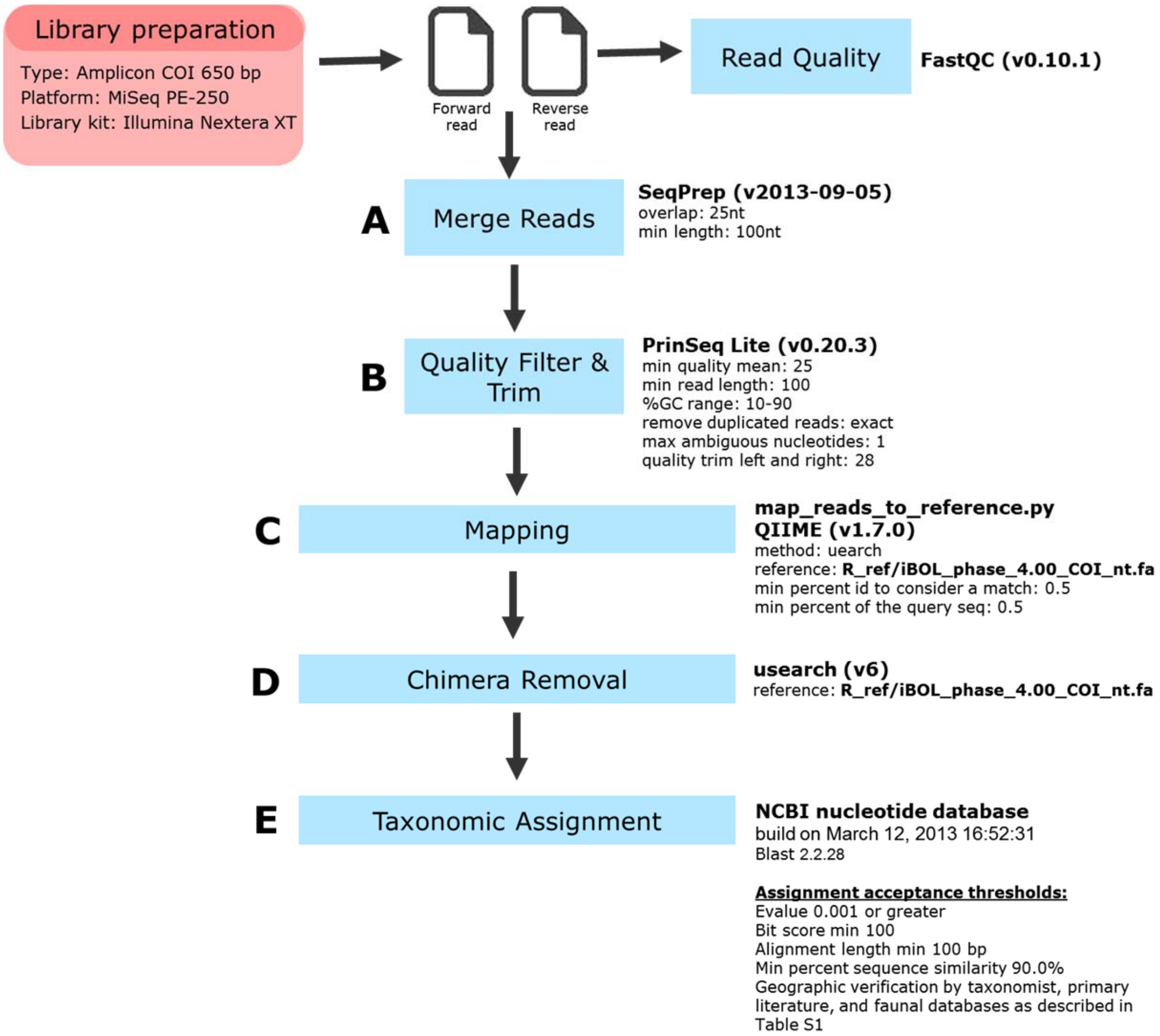

Supplementary Figure 1: Bioinformatic workflow for each step of filtering and parameters used in the various programs. Program citations are in the methods section. Minimum is abbreviated ‘min’ and base pair is abbreviated ‘bp’.

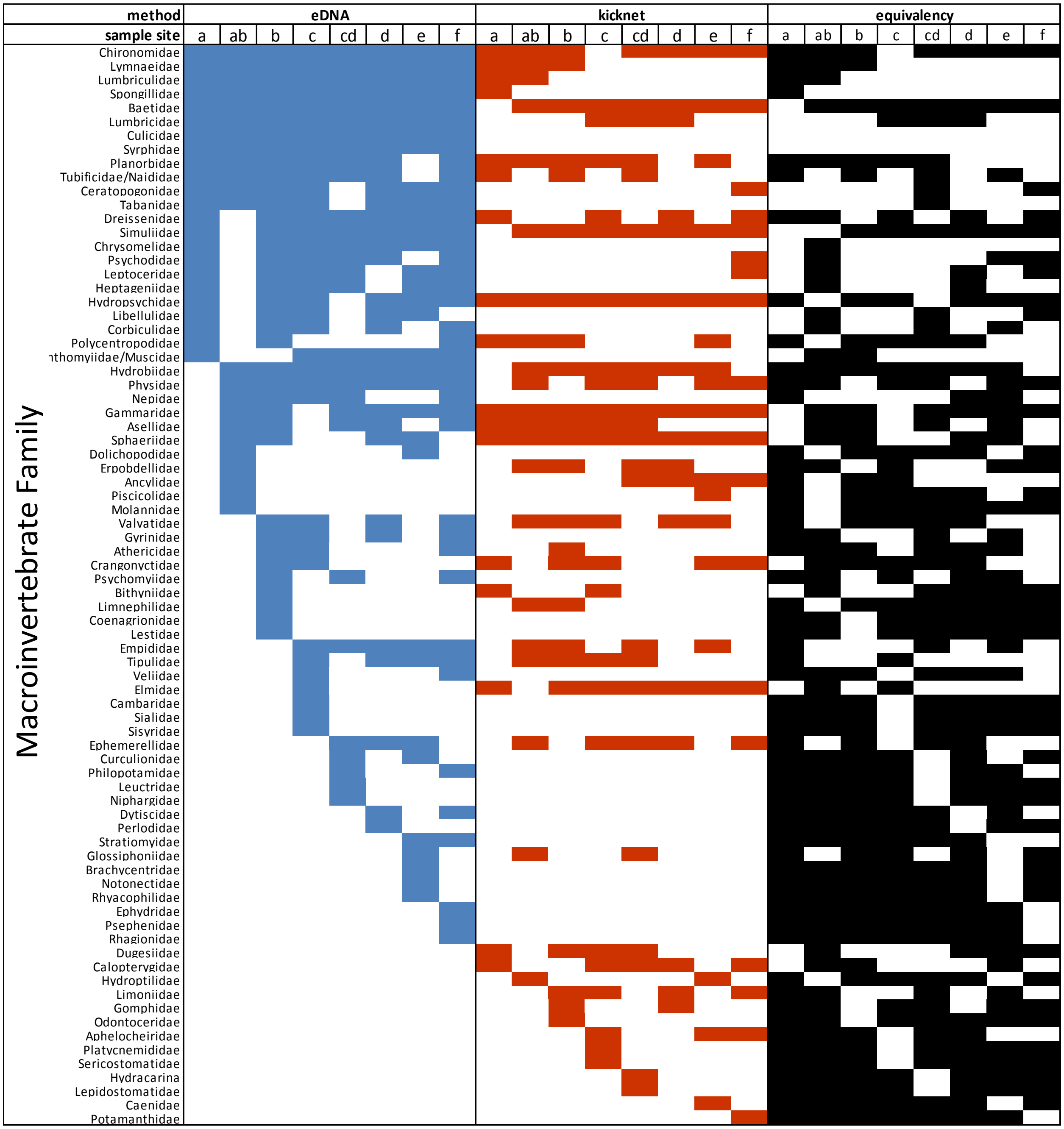

Supplementary Figure 2: Heatmap illustrating each macoinvertebrate family, its detection across each site and the equivalency in detection for environmental DNA and kicknet sampling methods. Blue indicates presence for eDNA, red indicates presence for kicknet and white indicates not detected. For the equivalency, black indicates both were or were not detected at the same site, white indicates the family was detected by only one of the sampling methods.

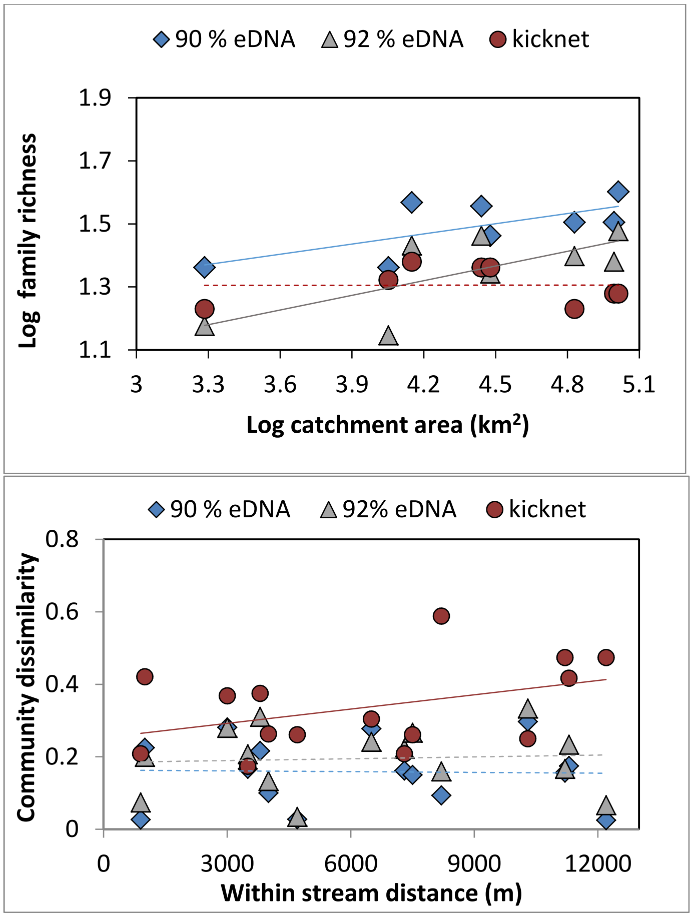

Supplementary Figure 3: Figure 2 from main text, but now depicted at two levels of bioinformatic stringency applied to eDNA metabarcoding estimated richness (i.e., 90 % similarity in assignment and 92 % similarity in assignment used as threshold for accepting a taxonomic name). a) Change in species richness relative to catchment area. Both axes were log transformed to illustrate their linear relationship. b) Change in beta diversity with respect to steam distance. All beta values for the three response variables (90 % eDNA, 92 % eDNA and kicknet) were recalculated using β_{-2} for estimating beta diversity instead of the Jaccard method for estimating beta diversity because this is more appropriate given the false negative signal generated by the removal of data.

Supplementary Table 1: Macroinvertebrate families that were captured in kicknet samples, but were not considered detected by environmental DNA due to bioinformatic thresholds set for accepting an assignment

Family	# Genbank sequences	a	ab	b	c	cd	d	e	f	Total	Average % similarity	Average assignment length
Aphelocheiridae	12									0	NA	NA
Caenidae	277		22					6		28	76.4	213.5
Calopterygidae	381			1				1		2	94.1	42.0
Dugesiidae	204	24		14	5		5	14	20	82	94.3	50.9
Gomphidae	680	1	29		6	1	7	1	9	54	90.3	67.8
Hydroptilidae	2081	513	3		4	3	3	3	76	605	86.8	105.5
Hydracarina	1394		1	1			24	3	6	35	93.9	113.6
Lepidostomatidae	844	27	3	21	8		5	10	9	83	83.2	82.2
Limoniidae	2053			1		1		1		3	82.1	132.7
Odontoceridae	263					2				2	81.8	103.5
Platycnemididae	153			2	3	1				6	82.3	145.2
Potamanthidae	6									0	NA	NA
Sericostomatidae	32		9		2	31				42	86.6	80.7

For each Macroinvertebrate family detected by the kicknet we list the number of sequences matching this family for cytochrome c oxidase I (COI) on Genbank (# Genbank sequences), the number of sequences that were identified but excluded at each site (a, ab, b, c, cd, d, e, and f) as well as their total across all eight site. We report the average similarity of the sequence matches to Genbank sequences, and the average assignment length of these sequences per family. Based on accepting assignments when these two variables met the criteria of a length greater than 100 bp and percent similarity greater than 90 %, Therefore, we have evidence that at least 11 of the 13 families found in the kicknet were not considered detected even though they exist in our dataset. This represents our known our false absence rate under the current bioinformatic thresholds.

Supplementary Table 2: Macroinvertebrate families that were considered detected by environmental DNA, but not detected in our 2012 kicknet sample

**Family**	**a**	**ab**	**b**	**c**	**cd**	**d**	**e**	**f**	**Greifensee**	**Total # Individuals**
Anthomyiidae/Muscidae										0
Brachycentridae										0
Cambaridae	1								3	4
Chrysomelidae										0
Coenagrionidae			17			1			2	20
Corbiculidae			1							1
Culicidae										0
Curculionidae										0
Dolichopodidae										0
Dytiscidae		1							1	2
Ephydridae										0
Gyrinidae				1						1
Heptageniidae										0
Lestidae										0
Leuctridae										0
Libellulidae										0
Molannidae									1	1
Nepidae										0
Niphargidae										0
Notonectidae									1	1
Perlodidae										0
Philopotamidae										0
Psephenidae										0
Psychomyiidae	5			8				5	9	27
Rhagionidae										0
Rhyacophilidae	100		20	11		21				152
Sialidae			1						2	3
Sisyridae										0
Stratiomyidae										0
Syrphidae										0
Tabanidae										0
Veliidae										0

Numbers recorded for each site (a, ab, b, c, cd, d, e, and f) as well as in lake Greifensee (of which is the headwaters for the river Glatt) represent individuals that were sampled by kicknet samples in previous years of monitoring (1995 - 2011)

Supplementary Table 3: The 33 taxon used to construct mock community.

Morphological Assignment					
**Phylum**	**Class**	**Order**	**Family**	**Habitat**	**DNA conc (ng/μL)**
Annelida	Clitellata	Arhynchobdellida	Erpobdellidae	aquatic	33.8
Annelida	Clitellata	Haplotaxida	Lumbricidae	terrestrial	49.0
Annelida	Clitellata	Rhynchobdellida	Glossiphoniidae	aquatic	40.9
Arthropoda	Arachnida	Trombidiformes	Hygrobatidae	aquatic	3.3
Arthropoda	Insecta	Coleoptera	Coccinellidae	terrestrial	36.0
Arthropoda	Insecta	Coleoptera	Elmidae	aquatic	6.8
Arthropoda	Insecta	Diptera	Chironomidae	aquatic	4.4
Arthropoda	Insecta	Diptera	Drosophilidae	terrestrial	3.4
Arthropoda	Insecta	Diptera	Simuliidae	aquatic	33.0
Arthropoda	Insecta	Diptera	Tabanidae	aquatic	9.1
Arthropoda	Insecta	Diptera	Tipulidae	aquatic	35.4
Arthropoda	Insecta	Ephemeroptera	Baetidae	aquatic	14.6
Arthropoda	Insecta	Ephemeroptera	Ephemerellidae	aquatic	21.8
Arthropoda	Insecta	Ephemeroptera	Heptageniidae	aquatic	17.3
Arthropoda	Insecta	Hymenoptera	Formicidae	terrestrial	4.7
Arthropoda	Insecta	Megaloptera	Sialidae	aquatic	18.7
Arthropoda	Insecta	Plecoptera	Capniidae	aquatic	12.8
Arthropoda	Insecta	Plecoptera	Leuctridae	aquatic	10.3
Arthropoda	Insecta	Trichoptera	Hydropsychidae	aquatic	9.2
Arthropoda	Insecta	Trichoptera	Limnephilidae	aquatic	39.4
Arthropoda	Insecta	Trichoptera	Philopotamidae	aquatic	10.3
Arthropoda	Insecta	Trichoptera	Psychomyiidae	aquatic	4.8
Arthropoda	Insecta	Trichoptera	Rhyacophilidae	aquatic	12.3
Arthropoda	Malacostraca	Amphipoda	Crangonyctidae	aquatic	19.8
Arthropoda	Malacostraca	Amphipoda	Gammaridae	aquatic	79.5
Arthropoda	Malacostraca	Amphipoda	Niphargidae	aquatic	9.0
Arthropoda	Malacostraca	Decapoda	Cambaridae	aquatic	60.2
Arthropoda	Malacostraca	Isopoda	Asellidae	aquatic	8.4
Mollusca	Bivalvia	Veneroida	Dreissenidae	aquatic	52.0
Mollusca	Gastropoda	Basommatophora	Planorbidae	aquatic	32.6
Mollusca	Gastropoda	Hygrophila	Lymnaeidae	aquatic	60.0
Mollusca	Gastropoda	Hygrophila	Physidae	aquatic	90.8
Mollusca	Gastropoda	Littorinimorpha	Hydrobiidae	aquatic	29.4

Supplementary Table 4: Taxonomic assignments of sequences to references compared with the assignment from blast searches against the NCBI nucleotide database

Reference Assignment							NCBI Assignment					
Phylum	Class	Order	Family	# sequences	Average of % similarity reference	Average of alignment length reference	Phylum2	Class2	Order2	Family2	Average of % similarity NCBI	Average of alignment length NCBI
Annelida	Clitellata	Rhynchobdellida	Glossiphoniidae	2	99.9	361.0	Annelida	Clitellata	Rhynchobdellida	Glossiphoniidae	92.5	360.0
Arthropoda	Insecta	Coleoptera	Coccinellidae	9571	99.5	312.4	Arthropoda	Insecta	Coleoptera	Coccinellidae	99.3	312.4
Arthropoda	Insecta	Diptera	Chironomidae	122	99.6	319.6	Arthropoda	Insecta	Diptera	Chironomidae	98.2	319.2
Arthropoda	Insecta	Diptera	Drosophilidae	88	99.4	315.5	Arthropoda	Insecta	Diptera	Drosophilidae	99.1	315.5
Arthropoda	Insecta	Diptera	Simuliidae	3897	98.4	302.6	Arthropoda	Insecta	Diptera	Simuliidae	94.7	301.5
Arthropoda	Insecta	Diptera	Tabanidae	1043	99.4	304.3	Arthropoda	Insecta	Diptera	Tabanidae	93.2	302.6
Arthropoda	Insecta	Diptera	Tabanidae	6	99.5	247.7	Arthropoda	Insecta	Diptera	Pipunculinae	91.7	246.0
Arthropoda	Insecta	Diptera	Tabanidae	5	98.9	246.3	Arthropoda	Insecta	Diptera	Sepsidae	92.1	241.7
Arthropoda	Insecta	Diptera	Tabanidae	1	99.2	245.0	Arthropoda	Insecta	Diptera	Simuliidae	91.0	245.0
Arthropoda	Insecta	Ephemeroptera	Baetidae	12719	99.7	333.6	Arthropoda	Insecta	Ephemeroptera	Baetidae	99.6	333.5
Arthropoda	Insecta	Ephemeroptera	Ephemerellidae	14	99.7	327.0	Arthropoda	Insecta	Ephemeroptera	Ephemerellidae	91.7	312.6
Arthropoda	Insecta	Ephemeroptera	Heptageniidae	977	99.8	323.7	Arthropoda	Insecta	Ephemeroptera	Heptageniidae	97.5	323.4
Arthropoda	Insecta	Hymenoptera	Formicidae	368	99.8	307.3	Arthropoda	Insecta	Hymenoptera	Formicidae	97.6	306.7
Arthropoda	Insecta	Megaloptera	Sialidae	9234	99.8	321.4	Arthropoda	Insecta	Megaloptera	Sialidae	98.9	321.3
Arthropoda	Insecta	Plecoptera	Capniidae	686	99.6	319.5	Arthropoda	Insecta	Plecoptera	Capniidae	93.4	317.6
Arthropoda	Insecta	Plecoptera	Leuctridae	3	99.6	243.0	Arthropoda	Insecta	Diptera	Chironomidae	90.3	229.3
Arthropoda	Insecta	Plecoptera	Leuctridae	3	99.6	240.0	Arthropoda	Insecta	Diptera	Lonchaeidae	90.4	229.5
Arthropoda	Insecta	Plecoptera	Leuctridae	1	96.4	247.0	Arthropoda	Insecta	Diptera	Tabanidae	90.3	247.0
Arthropoda	Insecta	Plecoptera	Leuctridae	1	97.4	302.0	Arthropoda	Insecta	Lepidoptera	Notodontidae	90.3	267.0
Arthropoda	Insecta	Trichoptera	Limnephilidae	382	99.7	299.7	Arthropoda	Insecta	Trichoptera	Limnephilidae	90.4	297.3
Arthropoda	Insecta	Trichoptera	Philopotamidae	553	99.7	259.0	Arthropoda	Insecta	Trichoptera	Philopotamidae	90.5	244.0
Arthropoda	Insecta	Trichoptera	Psychomyiidae	3	99.9	363.5	Arthropoda	Insecta	Trichoptera	Psychomyiidae	90.1	359.5
Arthropoda	Malacostraca	Amphipoda	Crangonyctidae	10	99.7	331.2	Arthropoda	Malacostraca	Amphipoda	Crangonyctidae	99.8	329.1
Arthropoda	Malacostraca	Amphipoda	Gammaridae	1	99.6	261.0	Arthropoda	Malacostraca	Amphipoda	Gammaridae	94.6	261.0
Arthropoda	Malacostraca	Decapoda	Cambaridae	13	99.4	309.5	Arthropoda	Malacostraca	Decapoda	Cambaridae	97.6	307.6
Arthropoda	Malacostraca	Isopoda	Asellidae	95	99.8	311.7	Arthropoda	Malacostraca	Isopoda	Asellidae	97.4	311.4
Mollusca	Bivalvia	Veneroida	Dreissenidae	388	99.8	323.4	Mollusca	Bivalvia	Veneroida	Dreissenidae	99.1	323.4
Mollusca	Gastropoda	Basommatophora	Planorbidae	4	99.2	329.2	Mollusca	Gastropoda	Basommatophora	Planorbidae	92.3	328.5
Mollusca	Gastropoda	Hygrophila	Lymnaeidae	7208	99.8	308.6	Mollusca	Gastropoda	Hygrophila	Lymnaeidae	91.8	301.9
Mollusca	Gastropoda	Hygrophila	Physidae	9245	99.7	315.8	Mollusca	Gastropoda	Hygrophila	Physidae	97.9	311.0
Mollusca	Gastropoda	Littorinimorpha	Hydrobiidae	998	99.8	324.8	Mollusca	Gastropoda	Littorinimorpha	Hydrobiidae	98.5	324.5

Grey bars inserted to align with taxa not detected from Supplementary Table 3.

Supplementary Table 5: Number of taxa recovered and sequences assigned correctly or incorrectly relative to the reference sequences used in the mock community

Taxon level	# taxa	% of total
Correctly recovered and assigned	25	76
Not recovered	8	24
Incorrectly assigned	2	8
Total taxa in mock community	33	
**Sequence level**	**# sequences**	**% of total**
Correctly assigned	57621	99.97
Incorrectly assigned	20	0.03
Total Sequences after bioinformatic filtering	57641	

Supplementary Table 6: Annual bioassessment costs in millions of US dollars for freshwater resources

Target Group	USA[1]	England[2]	Switzerland[3]
Fish	31.4 - 58.2	NA	0.35 (0.33 CHF)
Benthic invertebrates	38.1 - 70.7	NA	0.61 (0.58 CHF)
Algae (diatoms)	34.7 - 64.5	NA	0.32 (0.3 CHF)
Macrophytes	NA	NA	0.3 (0.29 CHF)
Total	104.2 - 193.4	11.6 (7.3£)	1.58 (1.5 CHF)

Information not available is abbreviated as NA. Original currency in brackets. Sources: [1]; [2] Richard Walmsley, Forestry Commission personal communication; [3] Markus Wüest, Federal Office for the Environment FOEN personal communication

Supplementary Table 7: Description of sites and mock community FASTq file names for raw sequence data generated and used in this study

Site name	File name	MD5	BioSample name	Alias
A	02-01_S12_L001_R1_001.fastq	000d11b208acfbaa28c0c3ef9f36a4ed	SAMN03946218	Site A 02-01_S12
A	02-01_S12_L001_R2_001.fastq	572038d5e92ee7c465d9ebe16b9f5af5	SAMN03946218	Site A 02-01_S12
AB	08-01_S18_L001_R1_001.fastq	3715cf40a0ffabd3bdc3ebdec622e7f9	SAMN03946320	Site AB 08-01_S18
AB	08-01_S18_L001_R2_001.fastq	6a501fc3fc84a379219667e5bf03a279	SAMN03946320	Site AB 08-01_S18
B	07-01_S17_L001_R1_001.fastq	fde4163fe9c00b89b449d3fb735adfd8	SAMN03946319	Site B 07-01_S17
B	07-01_S17_L001_R2_001.fastq	b9eb9d61c2f49a32b958507dc65a18e9	SAMN03946319	Site B 07-01_S17
C	03-01_S13_L001_R1_001.fastq	40c15149f687d7d8d5716d575785c838	SAMN03946321	Site C 03-01_S13
C	03-01_S13_L001_R2_001.fastq	1de1051f38598c17f59fe361cc6967d4	SAMN03946321	Site C 03-01_S13
CD	01-01_S11_L001_R1_001.fastq	1581c18f4e689458b97b992f61f9bb33	SAMN03946322	Site CD 01-01_S11
CD	01-01_S11_L001_R2_001.fastq	3102621c3a5731e412d14b12bd85336e	SAMN03946322	Site CD 01-01_S11
D	04-01_S14_L001_R1_001.fastq	549907979e502830c1aab1fc4cca0024	SAMN03946323	Site D 04-01_S14
D	04-01_S14_L001_R2_001.fastq	2f4344d766fc94f4065e3803f99dc770	SAMN03946323	Site D 04-01_S14
E	05-01_S15_L001_R1_001.fastq	1bcbab94440db04da4d65549d1e947b6	SAMN03946324	Site E 05-01_S15
E	05-01_S15_L001_R2_001.fastq	994a6a41011ecda2cf3071b61535d470	SAMN03946324	Site E 05-01_S15
F	06-01_S16_L001_R1_001.fastq	b3fb3f5547c7e1fcd861ece6939a9703	SAMN03946325	Site F 06-01_S16
F	06-01_S16_L001_R2_001.fastq	78f258c000c832e89e50ba9fc992c093	SAMN03946325	Site F 06-01_S16
MC1-4A	4A_S12_L001_R1_001.fastq	22348712e5534ebd00602a4363fc5961	SAMN05017775	MC PCR 1
MC1-4A	4A_S12_L001_R2_001.fastq	f30f9228f63884e48c81dcd739734439	SAMN05017775	MC PCR 1
MC2-4B	4B_S13_L001_R1_001.fastq	4703ccf6305ed2736e83fe1206fa03e4	SAMN05017775	MC PCR 2
MC2-4B	4B_S13_L001_R2_001.fastq	edacf5d3b6892d02fa15bcdb68498441	SAMN05017775	MC PCR 2
MC3-4D	4D_S15_L001_R1_001.fastq	252a9e5f38f4f98e2b2adbadc10442d7	SAMN05017775	MC PCR 3
MC3-4D	4D_S15_L001_R2_001.fastq	633e0dcc099394489f6526532a4694dc	SAMN05017775	MC PCR 3

Each file's MD5, Sequence Read Archive (SRA) accession number is listed for BioProject PRJNA291617 and the Alias for the sample names used in the SRA

Supplementary Note 1: Mock Community Analysis

Methods:

To assess both the robustness of the COI primers used in this study and to understand potential assignment error when using the NCBI nucleotide database we conducted a mock community analysis. The mock community was constructed from 33 families of metazoan eukaryotes spanning three phyla most prominently found in our study (Annelida, Arthropoda and Mollusca, Supplementary Table 3) and reflecting the taxa to be found in the traditional approach. These taxa also represent a diversity in aquatic and terrestrial habitat use. All taxa are from families we detect in our eDNA analysis. DNA was extracted from tissue using the DNeasy blood and tissue kit (Qiagen, Inc. Valencia, CA, USA) following the recommended manufacturer's protocol and quantified using the Qubit Broad Range dsDNA DNA Assay (Agilent Technologies, Santa Clara, CA, USA). Each taxon's DNA was then Sanger sequenced to generate a reference library following that of Mächler et al. [1]. 2 uL from each taxon at the concentration of their DNA extraction were subsequently pooled into a single mixture and was used as template for PCR and sequencing. We chose this method of pooling since in many cases the whole specimen was used for the extraction and thus DNA concentration is likely relative to body mass. PCR and library preparation followed exactly the protocol used for processing the environmental samples except that PCR replicates were not pooled and were separately indexed. Bioinformatics filtering of the raw reads followed exactly the same pipeline except reads at step C in the workflow (Supplementary Fig. 1) were mapped to the reference sequences. Sequences were then assigned using the blast algorithm to the newly generated reference sequences to ascertain the assignment length for each sequence and the percent similarity it had with the reference sequence. Sequences where then processed the same as for the environmental samples (steps D and E, Supplementary Fig. 1) and assigned taxonomic information based on the

exact NCBI nucleotide database build on March 12, 2013 16:52:31 used for all our other assignments in the manuscript. The resulting sequences were summarized across PCR replicates by their taxonomic assignments by totaling the number of sequences from any PCR replicate and taking the average for assignment statistics of these sequences. The same set of sequences were also compared against the tissue generated DNA sequences and their assignment were also summarized across PCR replicates by their taxonomic assignments by totaling the number of sequences from any PCR replicate and taking the average for assignment statistics of these sequences. (Supplementary Table 4).

Discussion:

The analysis of the mock community sheds light on the precision and accuracy of our primers to adequately assess biodiversity of metazoan eukaryotes. First, we recovered three fourths of the taxa pooled for the mock community assessment and recovered all three phyla inclusive of both aquatic and terrestrial taxa confirming our results from eDNA samples from a natural environment. These results provide strong support that our recovered taxa are not due to contamination in the laboratory setting or from bioinformatic assignment artifacts and that our workflow is capable of recovering taxa that are known to be present. However, several taxa were missed and this could be due to many reasons, such as primer bias [2]. Therefore, it is clear that a single primer set is not going to achieve full accuracy for description of DNA found in an eDNA sample, as is discussed in the main manuscript.

We did detect some assignment errors when comparing assignments of sequences to the reference sequence generated from the tissue extracted DNA to that of comparisons to the NCBI nucleotide database. Specifically, we used the exact criteria used to assign taxonomic information to our eDNA generated sequences by assigning the NCBI's top hit with the thresholds set for including a

taxonomic assignment (bit score of 100, alignment length greater than 100 bp and greater than 90 % similarity). The error in assignment of any sequence was extremely low at 0.03% of the total sequences analyzed and from a taxonomic perspective resulted in an 8% (2/25) error rate (Supplementary Table 5).

However, this rate of miss-assignment is very low when compared to error rates reported from classic identifications: morphological identification of specimens from a similar taxonomic list of macroinvertebrates is reported to vary between 22.1 and 33.8 % at the family level between identifiers from different laboratories [3,4]. Therefore, in comparison our sequence based identification has already the potential to improve identification error rates by two to four-fold. The difference in the average percent similarity in assignment at the family level between the reference sequence and NCBI's nucleotide database signifies there are gaps in the database (see also discussion highlighting this aspect in the main manuscript). Furthermore, using a 90% similarity threshold was able to achieve a correct assignment to the family level for three mock community taxa (Limnephilidae, Philopotamidae, Psychomyiidae, (Supplementary Table 4) indicating that increasing this stringency would create just as many false negative assignments as that for false positive assignments.

Thus, we have likely achieved the best possible balance between type I and type II error. We have added the above points to the discussion in the main manuscript in support of these observations from our mock community analysis.

Supplementary References

1 Mächler, E., Deiner, K., Steinmann, P. & Altermatt, F. Utility of Environmental DNA for Monitoring Rare and Indicator Macroinvertebrate Species. *Freshwater Science* **33**, 1174-1183, doi:10.1086/678128 (2014).

2 Elbrecht, V. & Leese, F. Can DNA-based ecosystem assessments quantify species abundance? Testing primer bias and biomass-sequence relationships with an innovative metabarcoding protocol. *PeerJ PrePrints* **3**, e1258 (2015).

3 Haase, P., Pauls, S. U., Schindehütte, K. & Sundermann, A. First audit of macroinvertebrate samples from an EU Water Framework Directive monitoring program: human error greatly lowers precision of assessment results. *Journal of the North American Benthological Society* **29**, 1279-1291, doi:10.1899/09-183.1 (2010).

4 Stribling, J. B., Pavlik, K. L., Holdsworth, S. M. & Leppo, E. W. Data quality, performance, and uncertainty in taxonomic identification for biological assessments. *Journal of the North American Benthological Society* **27**, 906-919, doi:10.1899/07-175.1 (2008).
